# Mechanisms and Definitions of Periprocedural Myocardial Infarction in the Era of Modern Revascularization

**DOI:** 10.31083/j.rcm2310351

**Published:** 2022-10-18

**Authors:** Arnaud Ferrante, Paul Guedeney, Johanne Silvain, Michel Zeitouni, Jean-Philippe Collet

**Affiliations:** ^1^Sorbonne Université, ACTION Study Group, INSERM UMRS_1166 Institut de cardiologie, Pitié Salpêtrière (AP-HP), 75013 Paris, France

**Keywords:** periprocedural myocardial infarction, percutaneous coronary intervention, coronary artery bypass graft

## Abstract

One of the most frequent complications following coronary revascularization is cardiac myonecrosis characterized by an elevation of cardiac biomarkers, particularly with the implementation of high-sensitivity cardiac troponin. In the last decades, various definitions of periprocedural myocardial injury and infarction have been proposed, based on different cardiac biomarkers, various thresholds, and the need for additional ischemic features. In this review, we aim at providing insights on the mechanisms involved in periprocedural myocardial injury and infarction following percutaneous coronary intervention or coronary artery bypass grafting, the strengths and limitations of the available definitions and their clinical implications. We also provide an updated description of preventive strategies that have been evaluated in randomized controlled trials to avoid these complications as well as patient-level and lesion-level risk factors to better anticipate and rebalance the indication for coronary revascularization and plan adequate post-procedure monitoring.

## 1. Introduction 

Percutaneous coronary intervention (PCI) has progressively become the primary means of coronary revascularization and is considered a safe procedure with low rates of major procedural complications, and which can be performed even in ambulatory conditions. Cardiac myonecrosis characterized by an elevation of cardiac biomarkers, particularly with the implementation of high-sensitivity cardiac troponin (hs-cTn) remains the most frequent complication of PCI [1]. Periprocedural myocardial injury and infarction are essentially distinguished by the magnitude of cardiac biomarker elevation, and how to best define such complications has been the subject of intense debate as their impact on the future occurrence of major cardiovascular events (MACE) and long-term mortality may significantly vary according to the definition used. Periprocedural myocardial infarction (MI) is also called “MI associated with percutaneous coronary intervention” or “type 4a MI”, while “type 4b MI” corresponds to MI caused by stent thrombosis and “type 4c MI” to infarctions related to in-stent restenosis [2].

Several academic groups have provided expert consensus-based definitions of periprocedural myocardial injury and infarction, including different biomarkers such as creatine kinase myocardial band (CK-MB) or cardiac troponin (cTn), varying thresholds, and the requirement or not for cardiac imaging evaluation, leading to significant variations in terms of sensitivity and specificity. According to the selected definition, recent studies have reported highly variable incidence rates of periprocedural myocardial injury or infarction [2, 3, 4, 5, 6], which were associated [1, 3] or not [7, 8] with MACE and long-term mortality.

Establishing a consensual definition is of importance, as periprocedural myocardial infarction is commonly used as a component of the primary composite endpoint of major clinical trials comparing coronary revascularization methods (i.e., PCI versus coronary artery bypass grafting) and the choice of definition has been demonstrated to influence both the outcomes and the clinical meaning of observed statistical differences, causing some controversy in the medical community [9, 10, 11, 12].

In this review, we detail and compare the most frequently used definitions, and provide insights on risk factors of periprocedural myocardial injury and infarction as well as documented preventive strategies.

## 2. Mechanisms 

Different mechanisms such as acute side branch occlusion (SBO), distal embolization, abrupt vessel closure (mainly secondary to acute thrombosis or dissection), vasospasm or slow-flow/no-reflow phenomenon can be involved in the occurrence of PCI complications, with multifactorial pathophysiological pathways [13, 14].

Acute side branch occlusion is the most commonly reported cause of periprocedural myocardial infarction (MI) in PCI [15] and may result from a plaque shift or an embolization from the main vessel into the side branch, an acute thrombosis or a dissection in the side branch, or a vasospasm involving a side branch [16, 17]. It is characterized by late gadolinium enhancement (LGE) adjacent to the stent using cardiovascular magnetic resonance (CMR). Its impact on outcome depends on the importance of the occluded side branch. The risk of SBO is increased in case of ostial stenosis of the side branch before stenting, a side branch origin within the primary arterial lesion, a small diameter of the side branch, a high balloon-to-artery ratio, the target segment, the stent type and complex procedures such as chronic total occlusion or atherectomy [18, 19, 20, 21, 22, 23, 24, 25].

The second most frequent cause of type 4a MI is a distal embolization of thrombus or atheromatous material, resulting in a slow flow or no reflow phenomenon, imaged by CMR as new LGE distal to the stent. Some strategies are associated with a lower risk of such phenomenon, including primary stenting, avoidance of high-pressure balloon or stent inflation or thrombectomy [26]. Intravascular imaging may also be useful to identify lesion at risk of embolization, by showing a thin-cap fibroatheromas by optical coherence tomography (OCT) or a significant plaque burden by intravascular ultrasound (IVUS) [27].

Abrupt vessel closure can also occur, usually caused by acute thrombosis, dissection, vasospasm, balloon-induced ischemia or even air embolism.

A slow flow or no reflow phenomenon can also be involved, related to distal embolization, loss of capillary autoregulation or microvascular spasm, all resulting in endothelial dysfunction [28]. Well-known cardiovascular risk factors, such as diabetes mellitus, hypertension, active smoking, dyslipidemia, kidney failure or inflammatory processes may also be associated with no reflow [29]. Use of longer stents or a high SYNTAX score II are associated with a higher risk of slow flow or no reflow phenomenon as well [29, 30].

Apart from these causes, a post-PCI elevation of biomarkers without an identifiable cause may still occur in nearly 20% of cases [16].

## 3. Diagnosis

High-sensitivity cardiac troponin is the most sensitive and specific cardiac biomarker for the diagnosis of periprocedural myocardial injury and type 4a MI [31]. Baseline (pre-PCI) and post-PCI cTn values should be routinely measured at 3–6 h post-PCI to detect such complications [2, 14]. In case of periprocedural flow-limiting complications or following rising post-PCI cTn values, further blood samplings at 12–24 h post-procedure may be considered to document the peak cTn values or confirm the diagnosis of type 4a MI [14]. Of note, baseline values of cTn are needed to correctly analyze any post-PCI elevations as chronic elevations may be present in about 30% of patients because of comorbidities and risk factors [32].

An electrocardiogram (ECG) after PCI is required to detect new ischemic changes: new ST-elevation at the J-point, new horizontal or downsloping ST-depression in two contiguous leads, new pathological Q waves or ST-elevation ≥1 mm concordant with the QRS in patients with left bundle branch block (LBBB) [2].

Transthoracic echocardiography (TTE) is also useful by showing new loss of viable myocardium or new regional wall motion abnormality, with the use of tissue Doppler imaging, speckle tracking or contrast agents to further improve sensibility, if needed. A TTE should be performed in patients with diagnosis of type 4a MI or with post-PCI cTn elevation of ≥5x 99th percentile upper reference limit (URL) within 48 h post-procedure [2, 14].

Presence of LGE by CMR is currently the gold-standard for the diagnosis and quantification of myocardial injury, as it can detect a mass of new irreversible myocardial injury from 0.8 to 5 g [33, 34, 35]. The amount of myocardial injury diagnosed by CMR directly correlates with post-PCI elevation of biomarkers [34] and the increased risk of MACE [33]. Two different patterns of LGE are described: new LGE adjacent to the stent, related to a side-branch occlusion, or distal to the stent, related to a distal embolization [34]. However, its use is highly restricted due to its limited availability in daily clinical practice.

Coronary angiography can show periprocedural flow-limiting complications such as coronary dissection, occlusion of a major epicardial artery, side branch occlusion or thrombus, disruption of collateral flow, or distal embolization. Intravascular imaging should be considered to better identify mechanical factors that may be responsible for coronary dissection or stent thrombosis and to clarify the pathophysiology of complications [36]. There is not always a correlation between the complications observed during the coronary angiogram and post-PCI cardiac biomarkers variations, as large complications may be associated with a non-significant biomarker elevation, while slight elevations in cardiac biomarkers may be noticed in the absence of any obvious angiographic complication. The coronary angiogram has to be carefully reviewed for subtle complications in patients with post-PCI cTn elevation of ≥5x 99th percentile URL within 48h post-procedure [14].

**Fig. 1.**
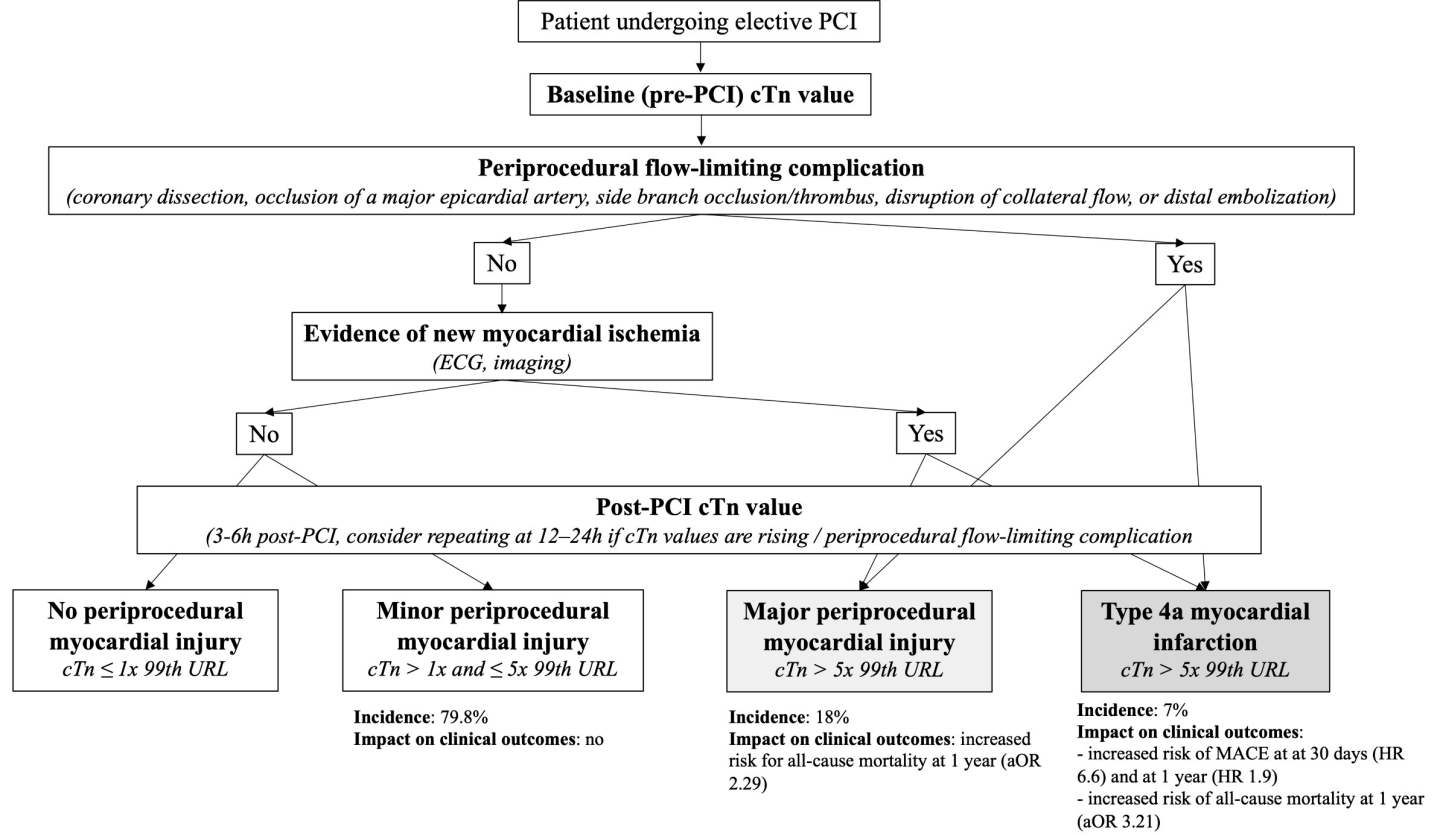
**Diagnostic algorithm for periprocedural myocardial injury or infarction**. Adapted from Bulluck *et al*. [37]. cTn, cardiac troponin; ECG, electrocardiogram; MACE, major adverse cardiovascular events; PCI, percutaneous coronary intervention; URL, upper reference limit.

A diagnostic algorithm for periprocedural myocardial injury or infarction has been proposed in a consensus document of the European Association of Percutaneous Cardiovascular Interventions (EAPCI) [14] (Fig. 1, Ref. [37]).

## 4. Definition of Periprocedural Injury and Myocardial Infarction

Over the past few years, scientific societies have published consensus statements attempting to standardize the definition of periprocedural myocardial injury and infarction (Table 1, Ref. [2, 13, 14, 38]). The main differences between these definitions are related to the type of biomarker considered, the diagnostic thresholds of theses biomarkers, and the place of additional features of ischemia (Table 2, Ref. [2, 13, 14, 38]).

**Table 1.** **Definitions of periprocedural myocardial injury and infarction**.

	Expert Consensus Document From the Society for Cardiovascular Angiography and Interventions (2014) [13]	The Academic Research Consortium-2 Consensus Document (2018) [38]	Fourth Universal Definition of Myocardial Infarction (2018) [2]	Consensus Document of the ESC Working Group on Cellular Biology of the Heart and European Association of Percutaneous Cardiovascular Interventions (2021) [14]
Periprocedural myocardial injury	Not mentioned	Absolute rise in cardiac troponin (from baseline) ≥70x URL	In patients with normal baseline values: elevation of cTn values >99th percentile URL	Minor (or no) periprocedural myocardial injury:
				elevation of cTn values within the first 48 hours following PCI >1 but ≤5x the 99th percentile URL
				no evidence of new myocardial ischemia (angiographic, imaging, electrocardiographic)
			In patients with abnormal baseline values but stable or falling: rise of cTn values >20% of the baseline value	Major periprocedural myocardial injury:
				elevation of cTn values within the first 48 hours following PCI >5x the 99th percentile URL
				no evidence of new myocardial ischemia (angiographic, imaging, electrocardiographic)
Myocardial infarction	In patients with normal baseline values, elevation of CK-MB values within the first 48 hours following PCI :	Absolute rise in cardiac troponin (from baseline) ≥35x URL	Elevation of cTn values within the first 48 hours following PCI :	Elevation of cTn values within the first 48 hours following PCI :
	≥10x ULN	Plus 1 (or more) of the following criteria:	>5x the 99th percentile URL in patients with normal baseline values	>5x the 99th percentile URL in patients with normal baseline values
	or ≥5x ULN + new pathologic Q-waves in ≥2 contiguous leads or new persistent LBBB	new significant Q waves or equivalent	rise >20% to an absolute value >5x the 99th percentile URL in patients with abnormal baseline values but stable (≤20% variation) or falling	rise >20% to an absolute value >5x the 99th percentile URL in patients with abnormal baseline values but stable (≤20% variation) or falling
	if CK-MB unavailable:	flow-limiting angiographic complications	Evidence of new myocardial ischemia:	Evidence of new myocardial ischemia :
	cTn (I or T) ≥70x ULN	new “substantial” loss of myocardium on imaging	new ischemic ECG changes	new ischemic ECG changes
	or cTn (I or T) ≥35x ULN + new pathologic Q-waves in ≥2 contiguous leads or new persistent LBBB	NB: applies to patients with baseline cTn levels <URL and to those in whom baseline cTn levels are elevated and stable or falling (when the baseline cTn is elevated and rising or when a second determination is superfluous (e.g., ST-segment–elevation MI), the ARC considers that it is not possible to reliably distinguish whether a subsequent biomarker rise results from the index MI or is a new MI related to a peri-procedural complication)	development of new pathological Q waves	development of new pathological Q waves
	In patients with elevated baseline values that are stable or falling, elevation of CK-MB values within the first 48 hours following PCI:		imaging evidence of new loss of viable myocardium or new regional wall motion abnormality in a pattern consistent with an ischemic etiology	imaging evidence of new loss of viable myocardium or new regional wall motion abnormality in a pattern consistent with an ischemic etiology
	by an absolute increment of ≥10x ULN		angiographic (or post-mortem) findings consistent with a procedural flow-limiting complication: coronary dissection, occlusion of a major epicardial artery or a side branch occlusion/thrombus, disruption of collateral flow, slow flow or no-reflow, or distal embolization	angiographic (or post-mortem) findings consistent with a procedural flow-limiting complication: coronary dissection, occlusion of a major epicardial artery or a side branch occlusion/thrombus, disruption of collateral flow, slow flow or no-reflow, or distal embolization
	if CK-MB unavailable: cTn (I or T) elevation by an absolute increment of ≥70x ULN			
	In patients with elevated baseline values that are rising:			
	elevation of CK-MB values within the first 48 hours following PCI:			
	by an absolute increment of ≥10x ULN			
	if CK-MB unavailable: cTn (I or T) elevation by an absolute increment of ≥70x ULN			
	new ST-segment elevation or depression			
	signs consistent with a clinically relevant MI : new onset or worsening heart failure, sustained hypotension			

ARC, Academic Research Consortium; CK-MB, creatine kinase myocardial band; cTn, cardiac troponin; ECG, electrocardiogram; LBBB, left bundle branch block; MI, myocardial infarction; PCI, percutaneous coronary intervention; ULN, upper limit of normal; URL, upper reference limit.

**Table 2.** **Main differences between the definitions of periprocedural myocardial infarction**.

	Consensus Document From the Society for Cardiovascular Angiography and Interventions (2014) [13]	The Academic Research Consortium-2 Consensus Document (2018) [38]	Fourth Universal Definition of Myocardial Infarction (2018) [2]	Consensus Document of the ESC Working Group on Cellular Biology of the Heart and European Association of Percutaneous Cardiovascular Interventions (2021) [14]
	SCAI	ARC2	4th UDMI	
Biomarker used	CK-MB (troponin if unavailable)	hs-cTn	hs-cTn	hs-cTn
Threshold (in patients with normal baseline values)	CK-MB ≥10x ULN	cTn ≥35x URL	cTn >5x the 99th percentile URL	cTn >5x the 99th percentile URL
Additional features of ischemia/infarction	no (except in those with elevated baseline values that are rising)	ECG, angiography, or imaging	symptoms, ECG, angiography, or imaging	symptoms, ECG, angiography, or imaging
Patients with abnormal baseline values and rising	additional features: new ST-segment elevation or depression, signs consistent with a clinically relevant MI	not possible to conclude	not mentioned	not mentioned
Accuracy	more specific	more sensitive	more sensitive	more sensitive

CK-MB, creatine kinase myocardial band; cTn, cardiac troponin; ECG, electrocardiogram; hs-cTn, high-sensitivity cardiac troponin; MI, myocardial infarction; ULN, upper limit of normal; URL, upper reference limit.

Concerning the chosen biomarker, the Society of Cardiovascular Angiography and Interventions (SCAI) uses CK-MB, while the Universal Definition of Myocardial Infarction (UDMI), the Academic Research Consortium-2 (ARC-2) and the EAPCI prefer cardiac troponin. It has been demonstrated that hs-cTn is the more sensitive and specific to rule out the diagnosis of myocardial infarction but also to detect periprocedural myocardial injury and infarction, compared to CK-MB which might not be as readily available in some centers as the former [31, 39].

The diagnostic thresholds of biomarkers for myocardial injury vary from any post-PCI elevations with the UDMI and EAPCI definition to cTn >70x URL with the ARC-2 definition, while the SCAI definition does not specify it. Of note, the EAPCI introduces an interesting distinction between minor periprocedural myocardial injury, defined as any biomarker elevation and major periprocedural myocardial injury defined as post-PCI cTn value >5x 99th percentile. A recent pooled-analysis of patient-level data by Silvain *et al*. [37] reported that myocardial injury defined as any post-PCI elevation in high-sensitivity cTn >99th percentile URL occurred in almost 80% of patients and was not associated with all-cause mortality at 1 year. In this analysis, the optimal cut-off for post-PCI cTn elevation to predict 1-year mortality was found to be >5x 99th percentile URL, corresponding to the threshold used by the EAPCI and the 4th UDMI, which was present in 18.2% of the patients. Furthermore, the association between the occurrence of type 4a myocardial infarction and all-cause death at one year was robust (adjusted Odds ratio [aOR] 3.21, 95% confidence interval [CI] 1.42–7.27) confirming the additional prognostic value of new onset of ischemic features. Prevalence of periprocedural myocardial injury according to the ARC-2 or SCAI criteria is considerably lower, affecting ≤2% of the patients [37, 40].

Finally, the UDMI, ARC-2 and EAPCI routinely require associated clinical, echocardiographic, or angiographic findings to establish the diagnosis of periprocedural MI, whereas SCAI only requires these findings among patients with elevated baseline values that are further rising following PCI.

## 5. Definitions in Randomized Controlled Trials

Periprocedural myocardial infarction is one of the components of the primary composite endpoint of pivotal clinical trials. However, the use of different definitions across studies (Table 3, Ref. [41, 42, 43, 44, 45, 46]) have been demonstrated to influence the outcomes and the clinical impact of the observed statistical differences [9, 10, 11, 12]. This is critical in clinical trials comparing PCI with medical therapy or coronary artery bypass grafting (CABG) surgery for the treatment of multivessel or left main coronary artery disease [11, 47]. In the Xience Versus Coronary Artery Bypass Surgery for Effectiveness of Left Main Revascularization (EXCEL) trial, both the SCAI and UDMI definition of periprocedural MI were collected during the trial and the stringent SCAI definition was finally chosen once released. As detailed above, this definition is the only one driven by cardiac biomarker elevation alone (without requiring additional features of ischemia) except for patients with elevated baseline values that are rising and uses the same threshold for both PCI and CABG. The conclusion of the trial after 3 years and 5 years of follow-up was that PCI was not inferior to CABG according to the composite primary outcomes of death, MI (both spontaneous and periprocedural) and stroke in patients with left main coronary artery disease of low or intermediate anatomical complexity [41, 48]. In a post-hoc analysis, the authors demonstrated that using the 4th UDMI, the rate of periprocedural MI after PCI increased from 3.6% to 4.0%, while it was reduced from 6.1% to 2.2% with CABG [9, 11]. Similar observations were made for the Taxus Drug-Eluting Stent Versus Coronary Artery Bypass Surgery for the Treatment of Narrowed Arteries (SYNTAX) trial, where the rates of periprocedural MI according to the protocol definition (CK-MB peak >10% or CK-MB ≥5x ULN associated with ECG criteria of ischemia), the 4th UDMI or the SCAI definition varied from 2.7% to 3.0% and to 5.7% respectively in the PCI arm, and from 2.4% to 2.1% and to 16.5% respectively in the CABG arm [10]. Interestingly enough, the occurrence of periprocedural myocardial injury following CABG was not significantly associated with 10-year mortality after adjustment on confounders, as opposed to PCI. Post-CABG periprocedural MI defined by combining biomarkers elevations and ECG or imaging abnormalities significantly predicted all-cause mortality at 1 and 10 years, while definitions based on isolated enzyme elevation did not have significant correlation with survival. Of note, the wide discrepancies in the incidence rates of periprocedural myocardial infarction following CABG have not been limited to randomized controlled trials and have also been reported based on large real-world registries [49]. Significant and isolated cardiac enzyme elevation may be observed following CABG corresponding to global cardiac injury possibly subsequent to cardioplegia and without a specific epicardial coronary artery de novo lesion or loss of graft patency [47]. The clinical implications of such events seems less certain following CABG than after PCI [10, 49].

**Table 3.** **Definitions of periprocedural myocardial infarction in main clinical trials**.

	PCI	CABG
SYNTAX [43]	≤7 days after intervention	
	∙ ratio peak CK-MB/peak total CK >10% or CK-MB ≥5x ULN	
	∙*and* ECG criteria: new Q-waves in ≥2 contiguous leads	
PRECOMBAT [44]	≤7 days after intervention	
	∙ ratio peak CK-MB/peak total CK >10% or CK-MB ≥5x ULN	
	∙*and* ECG criteria: new Q-waves in ≥2 contiguous leads or new LBBB	
FREEDOM [45]	≤14 days after intervention	
	∙ CK elevation >2x ULN or CK-MB elevation	
	∙*and* ECG criteria: new Q-waves in ≥2 contiguous leads	
BEST [46]	≤48 h after intervention	
	∙ CK-MB ≥5x ULN	
	∙*and* ECG criteria: new Q-waves in ≥2 contiguous leads or new LBBB	
EXCEL [41]	≤72 h after intervention	
	∙ CK-MB >5x ULN *and*:	
	∘ ECG criteria: new Q-waves in ≥2 contiguous leads or new persistent LBBB	
	∘*or* angiographic findings : graft or native coronary artery occlusion or new severe stenosis with thrombosis and/or diminished epicardial flow	
	∘*or* imaging evidence: new loss of viable myocardium or new regional wall motion abnormality	
	*or*	
	∙ CK-MB >10x ULN	
ISCHEMIA primary definition [42]	≤48 h after intervention	∙ CK-MB >10x ULN or cTn >70x ULN *and*:
	∙ CK-MB >5x ULN or cTn >35x ULN (or rise >20% in subjects with elevated baseline values that are stable or falling) *and:*	∘ ECG criteria: new Q-waves in ≥2 contiguous leads or new persistent LBBB
	∘ ECG criteria: new ST segment elevation or depression in 2 contiguous leads, new Q-waves in ≥2 contiguous leads, or new persistent LBBB	∘*or* imaging evidence: new substantial wall motion abnormality (except septal and apical abnormalities)
	∘*or* angiographic findings: TIMI 0/1 flow in a major coronary artery or a side branch with reference vessel diameter ≥2.0 mm which had TIMI 2–3 flow at baseline, or TIMI 2 flow in a major coronary artery or a side branch with reference vessel diameter ≥3.0 mm which had TIMI 3 flow at baseline or Type C dissection or greater in the target vessel	*or*
	*or*	∙ CK-MB >15x ULN or cTn >100x ULN
	∙ CK-MB >10x ULN or cTn >70x ULN	
ISCHEMIA secondary definition [42]	≤48 h after intervention	∙ cTn >10x 99th percentile URL or CK-MB >10x ULN *and*:
	∙ cTn >5x 99th percentile URL or CK-MB >5x ULN (or rise >20% in subjects with elevated baseline values that are stable or falling) *and*:	∘ ECG criteria: new Q-waves in ≥2 contiguous leads or new persistent LBBB
	∘ symptoms suggestive of myocardial ischemia (≥20 min)	∘ angiographic findings: new graft or new native coronary artery occlusion
	∘*or* ECG criteria: new ST segment elevation or depression in 2 contiguous leads, new Q-waves in ≥2 contiguous leads, or new persistent LBBB	∘ imaging evidence: new loss of viable myocardium
	∘*or* angiographic findings: flow limiting complication, such as loss of patency of a side branch, persistent slow-flow or no re-flow, embolization, or Type C dissection or greater in the target vessel	*or*
	∘*or*imaging evidence: new loss of viable myocardium or new regional wall motion abnormality	∙ cTn >100x 99th percentile URL or CK-MB >15x ULN
	*or*	
	∙ cTn >70x 99th percentile URL	

BEST, Bypass Surgery Versus Everolimus-Eluting Stent Implantation for Multivessel Coronary Artery Disease; CABG, coronary artery bypass grafting surgery; CK, creatine kinase; CK, creatine kinase myocardial band; ECG, electrocardiogram; EXCEL, Evaluation of Xience Versus Coronary Artery Bypass Surgery for Effectiveness of Left Main Revascularization; FREEDOM, Comparison of Two Treatments for Multivessel Coronary Artery Disease in Individuals With Diabetes; ISCHEMIA, International Study of Comparative Health Effectiveness With Medical and Invasive Approaches; LBBB, left bundle branch block; PCI, percutaneous coronary intervention; PRECOMBAT, Bypass Surgery Versus Angioplasty Using Sirolimus-Eluting Stent in Patients With Left Main Coronary Artery Disease; SYNTAX, TAXUS Drug-Eluting Stent Versus Coronary Artery Bypass Surgery for the Treatment of Narrowed Arteries; ULN, upper limit of normal; URL, upper reference limit.

Interestingly in the International Study of Comparative Health Effectiveness With Medical and Invasive Approaches (ISCHEMIA) trial, two definitions of periprocedural MI were analyzed, the first one based on CK-MB in association with ECG and angiographic findings, the second one using cTn in association with symptoms, ECG, imaging or angiographic evidence of ischemia [42]. In this study, the rate of periprocedural MI at 6 months in the invasive group was 2.6% using the former definition and increased to 7.7% using the latter, emphasizing the impact of the definition used on the outcomes and the interpretation of the clinical trials. It should be noted that patients undergoing coronary revascularization presented with higher rates of periprocedural MI at 6 months but with lower risk of spontaneous MI at 4 years compared to patient treated conservatively. Importantly, the occurrence of periprocedural MI, using both definitions was not associated with all-cause or cardiovascular mortality, compared to spontaneous MI.

## 6. Risk Factors of Periprocedural Injury and Myocardial Infarction 

A number of patients features, lesions characteristics and periprocedural factors have been reported to be independently associated with the onset of periprocedural myocardial injury, and type 4a MI following PCI [1, 14, 16, 37] (Table 4, Ref. [14]).

**Table 4.** **Independent predictors of myocardial injury, type 4a MI and MACE**.

	Myocardial injury and type 4a MI	MACE
Patient level	∙ Advanced age	∙ Advanced age
	∙ Renal failure	∙ Renal failure
	∙ Preprocedural cardiac biomarker elevation	∙ Preprocedural cardiac biomarker elevation
	∙ Current congestive heart failure	∙ Current congestive heart failure
		∙ Peripheral vascular disease
		∙ Prior MI
		∙ Prior stroke
		∙ Diabetes mellitus
		∙ Ever smoked
		∙ COPD
		∙ Ejection fraction
Lesions level	∙ Multivessel/diffuse CAD	∙ Lesions of the left main
	∙ Left main disease	∙ Calcified lesions
	∙ Bifurcation lesions	∙ Saphenous vein graft lesions
Procedure	∙ Stent length	∙ Multivessel interventions
	∙ Stent diameter	∙ Stent length >30 mm
	∙ Number of stents	∙ Post-procedural bleeding
	∙ Multivessel PCI	
	∙ Retrograde approach for CTO	
	∙ Rotational atherectomy	

Adapted from Bulluck *et al*. [14]. CAD, coronary artery disease; COPD, chronic obstructive pulmonary disease; CTO, chronic total occlusion; MACE, major adverse cardiovascular event; MI, myocardial infarction; PCI, percutaneous coronary intervention.

Patient-related factors independently associated with myocardial injury and type 4a MI after PCI are age [37], renal failure [50], preprocedural cardiac biomarker elevation [50], and congestive heart failure [51]. These comorbidities are major signs of overall frailty which could explain the higher risk of myocardial injury.

Lesion characteristics that have been demonstrated to be independently associated with myocardial injury and type 4a MI after PCI are multivessel lesions [52], left main disease [53] and bifurcation lesions [50]. Indeed, as the corresponding coronary arteries frequently cover large portion of myocardium, the risk of myocardial injury increases accordingly, while intervention on bifurcation lesions may result in alteration of the flow in the side branch.

Finally, procedure-related factors independently associated with the occurrence of myocardial injury and type 4a MI after PCI are the use of longer and/or larger stents [37], a greater number of implanted stents (aOR 1.5, 95% CI 1.1–2.3 for a number of implanted stents ≥3) [1], multivessel PCI [51], complex procedures such as retrograde approach for chronic total occlusion [54] or use of rotational atherectomy [51]. Such procedures usually require multiple balloon inflations across pre- and post-dilatation of the lesion and stent implantation. Each inflation carries the risk of distal embolus, coronary artery dissection or side branch occlusion.

The identification of these factors before the procedure could help individualizing high risk patients, thus allowing early implementation of preventive strategies and/or close monitoring following the PCI.

## 7. Outcomes Following Periprocedural Myocardial Infarction 

As previously mentioned, the specific association between periprocedural myocardial infarction and adverse outcomes may significantly vary according to the considered definitions. However, periprocedural myocardial injury (as opposed to MI) has also been associated with various adverse outcomes such as readmission for acute coronary syndrome or heart failure (OR 3.3, 95% CI 1.1–8.8) [55], unplanned revascularization (aHR 1.40, 95% CI 1.04–2.06) or target vessel revascularization (aHR 1.90, 95% CI 1.06–3.38) [56], MACE at 30 days (aHR 3.8, 95% CI 1.9–6.9) and one year (aHR 1.7, 95% CI 1.1–2.6), as well as cardiac death at one year (aHR 7.66, 95% CI 3.64–16.11) [40] or at 3 years (aHR 4.93, 95% CI 1.92–12.69) [57]. This morbid association may be explained by the direct consequence of loss of myocardium but also by the fact that periprocedural myocardial injury and infarction more frequently occur in frail patients with more extensive atherosclerotic disease.

## 8. Preventive and Management Strategies

### 8.1 Prior to Procedure

Current guidelines recommend the use of aspirin and clopidogrel (600 mg loading dose, 75 mg daily dose) in patients undergoing elective PCI [36]. For dual antiplatelet therapy (DAPT)-naïve patients, it is recommended, if possible, to delay the PCI by almost 2 h or even to the next day, as a loading dose of clopidogrel acts within 2 to 6 hours. Otherwise, a loading dose with ticagrelor or crushed prasugrel which onset of action starts within 30 min, with clopidogrel given subsequently (600 mg loading dose, 75 mg daily dose) may be used. The Assessment of Loading With the P2Y12 inhibitor Ticagrelor or Clopidogrel to Halt Ischemic Events in Patients Undergoing Elective Coronary Stenting (ALPHEUS) trial randomly assigned 1910 patients undergoing elective high-risk PCI to receive either ticagrelor (180 mg loading dose, 90 mg twice daily subsequently for 30 days) or clopidogrel (300–600 mg loading dose, 75 mg daily subsequently for 30 days) [58]. High-risk PCI was defined as at least one of the following features: age >75 years, renal insufficiency, diabetes mellitus, overweight, acute coronary syndrome in the past 12 months, left ventricular ejection fraction <40% and/or prior episode of heart failure, multivessel disease, multiple stenting, left main stenting, bifurcation stenting, American College of Cardiology/American Heart Association (ACC/AHA) type B2 or C lesion, stenting of venous or arterial coronary graft. Ticagrelor was not superior to clopidogrel in reducing the composite primary outcome of PCI-related myocardial infarction (type 4a or b according the 3rd UDMI) or major myocardial injury (OR 0.97, 95% CI 0.80–1.17; *p* = 0.75), did not cause an increase in major bleeding (OR 2.51, 95% CI 0.48–13.0, *p* = 0.29), but did increase the rate of minor bleeding at 30 days (OR 1.54, 95% CI 1.12–2.11; *p* = 0.0070) [53]. Consistently, the Intensified Loading With prasugrel Versus Standard Loading With Clopidogrel in Invasive-treated Patients With Biomarker-negative Angina Pectoris (SASSICAIA) trial compared a pre-PCI loading dose of prasugrel to clopidogrel in 781 patient undergoing elective PCI. Of note, after PCI, all patients were treated with clopidogrel 75 mg/day and aspirin. The trial was prematurely terminated because of slower-than-expected recruitment and found a non-significant 10% relative reduction in the rate of procedural events in the prasugrel arm compared to clopidogrel (all-cause death, any MI including myocardial injury defined as isolated elevation of hs-cTn >3x ULN and periprocedural MI according to the 3rd UDMI, stent thrombosis, urgent revascularization and stroke within 30 days after PCI) [59].

Pre-PCI use of high-dose statins (atorvastatin 80 mg, rosuvastatin 40 mg) is useful in reducing PCI-related events, as demonstrated in several randomized control trials [60, 61] and a meta-analysis [62]. In the latter which included 14 randomized controlled trials and comprising 3368 patients undergoing PCI, high-dose rosuvastatin preloading demonstrated a benefit in reducing MACE (OR 0.42, 95% CI 0.29–0.61; *p *< 0.00001) and periprocedural MI (OR 0.40, 95% CI 0.25–0.63; *p *< 0.0001), in both stable patients and those experiencing an acute coronary syndrome.

The randomized controlled Effects of Acute Colchicine Administration Prior to Percutaneous Coronary Intervention (COLCHICINE-PCI) trial randomized 400 patients to receive high-dose of colchicine or placebo prior to PCI and found no reduction of the composite outcome of death, nonfatal myocardial infarction (defined as type 1 or type 4a MI according to the UDMI), and target vessel revascularization at 30 days (11.7% vs. 12.9%, *p* = 0.82) as well as the risk of SCAI-defined periprocedural MI (2.9% vs. 4.7%, *p* = 0.49) [63].

### 8.2 During Procedure

Cangrelor is useful to achieve a full platelet-inhibition within minutes after the start of infusion and has been shown to reduce periprocedural MI rates, according to the 2nd UDMI and the SCAI, in a substudy of the Clinical Trial Comparing Cangrelor to Clopidogrel Standard Therapy in Subjects Who Require Percutaneous Coronary Intervention (CHAMPION-PHOENIX) [64]. Consequently, cangrelor may be considered in patients who have not received P2Y12 receptor inhibitors (class IIb recommendation) [65].

Glycoprotein IIb/IIIa inhibitors may currently be considered in specific ‘bail-out’ situations such as high intraprocedural thrombus burden, slow-flow or no-flow phenomenon with occlusion of the stented coronary vessel [36]. Intracoronary vasodilators (calcium channel blockers, nitroglycerine, nitroprusside or adenosine) are also useful in case of vasospasm or no-reflow.

As previously reported, intravascular imaging may help predicting the risk of periprocedural MI by characterizing plaque composition and identifying lesions at high-risk of atherothrombotic embolization. Such lesions are characterized in OCT by a thin-capped fibroatheromas, a long lipid length, a large lipid arc and are more likely to protrude into the lumen [66]. In IVUS, a significant plaque burden with an important necrotic core volume may be associated with a higher occurrence of slow-flow phenomenon [67]. Finally, a more recent intracoronary imaging technique, near-infrared spectroscopy (NIRS) also identified a large lipid core burden as a marker for the future occurrence of coronary events and may represent an alternative of interest to predict the risk of periprocedural MI [27, 68, 69].

Distal embolic protection using a filter device has also been evaluated to prevent periprocedural MI and demonstrated a reduction in the occurrence of no-reflow phenomenon and serious cardiac adverse events in patients with lesion with high-risk features of embolization in IVUS [70]. This could represent an interesting option in selected patients.

The Double Kissing and Double Crush Versus Provisional T Stenting Technique for the Treatment of Unprotected Distal Left Main True Bifurcation Lesions: A Randomized, International, Multi-Center Clinical (DKCRUSH-V) trial reported significantly higher periprocedural biomarker release (defined as troponin I or T >5ULN) after the double kissing and double crush (DK crush) technique compared to the provisional T stenting with 11.3% vs. 4.1%, respectively, however no significant differences were observed in terms of periprocedural MI according to the SCAI definition [71]. To our knowledge, no bifurcation treatment technique has yet shown superiority in reducing the risk of periprocedural infarction [72].

### 8.3 Following PCI

In patients diagnosed with type 4a MI or major periprocedural injury, the EAPCI consensus document and European society of Cardiology (ESC) guidelines recommend optimizing pharmacotherapy for risk factors modifications associated with permanent lifestyle changes in order to reduce the future occurrence of MACE [14, 36, 73]. Whether these patients would benefit from the addition of a beta-blocker, or an angiotensin-converting enzyme inhibitor should be investigated in dedicated future studies.

## 9. Conclusions

Elevation of cardiac biomarkers following PCI is quite common in daily clinical practice particularly with the implementation of hs-cTn. Various definitions have been proposed in the last decades using different cardiac biomarkers and thresholds and resulting in a wide range of prevalence and associations with adverse outcomes. As dedicated randomized controlled trials have failed to demonstrate the benefit of the use of potent P2Y12 inhibitors to prevent the risk of periprocedural MI, the focus should be made on identifying patient-level and lesion-level risk factors beforehand to better anticipate and rebalance the indication, implement preventive strategies, and ensure adequate post-procedure monitoring.

## Abbreviations

ARC-2, Academic Research Consortium-2; CI, confidence interval; CK-MB, creatine kinase myocardial band; CMR, cardiovascular magnetic resonance; cTn, cardiac troponin; EAPCI, European Association of Percutaneous Cardiovascular Interventions; ECG, electrocardiogram; hs-cTn, high-sensitivity cardiac troponin; LGE, late gadolinium enhancement; MACE, major cardiovascular events; MI, myocardial infarction; PCI, percutaneous coronary intervention; SCAI, Society of Cardiovascular Angiography and Interventions; UDMI, Universal Definition of Myocardial Infarction; ULN, upper limit of normal; URL, upper reference limit.
